# “I didn’t see a sheep”: perspectives of lecturers and students at veterinary schools in Great Britain on learning about lameness in sheep

**DOI:** 10.3389/fvets.2023.1171853

**Published:** 2023-06-09

**Authors:** Rachel Clifton, Kate Bamford, Laura Green

**Affiliations:** School of Life Sciences, University of Warwick, Coventry, United Kingdom

**Keywords:** education, footrot, lameness, sheep, veterinary students

## Abstract

**Introduction:**

Great Britain has over 15 million ewes. Lameness is one of the top three most economically important diseases for the sheep industry, costing about £80 million per annum. The prevalence of lameness reduced from 10% to 5% between 2004 and 2013 but further reduction is unlikely because many farmers and agricultural students still believe in, and use, ineffective practices to control lameness. Unfortunately, many veterinary practitioners consider themselves insufficiently knowledgeable to work confidently with sheep farmers, and many sheep farmers agree with them. Another route to improve control of lameness is to ensure that all new veterinary graduates are competent to advise farmers.

**Methods:**

Our study investigated how veterinary students are taught about management of lameness in sheep. Ten lecturers from eight veterinary schools were interviewed, and 33 students from four veterinary schools participated in four focus groups; all were recorded, transcribed, and analysed using directed qualitative content analysis.

**Results:**

Teaching time and opportunities for students to gain clinical experience of lameness were very limited. Students were not confident they could diagnose causes of lameness and listed many practices, including ineffective ones, to manage footrot.

**Discussion:**

We conclude that GB veterinary students are graduating without evidence-based understanding and clinical experience necessary to advise farmers on management of lameness in sheep. Given the importance of lameness in sheep in GB we conclude that an alternative approach to education on lameness in sheep could help to ensure that new graduate veterinarians can contribute to control of lameness in sheep.

## 1. Introduction

Great Britain (GB) has over 15 million breeding ewes and is the largest lamb meat producer in Europe (1). Footrot causes lameness in sheep and reduces health, welfare, and productivity. Farmers in GB report that footrot is one of the most important diseases in their flock (2) and lameness costs the sheep industry £24 -£80 million *per annum* (3, 4).

The most effective treatment for footrot is systemic antibiotic treatment of lame sheep within 3 days of onset of lameness without trimming hoof horn, hereafter termed ‘prompt treatment’ (3, 5). Prompt treatment reduces flock prevalence of lameness to <2% (3, 6) and removes production losses (3). Other managements that contribute to reducing incidence of lame sheep are quarantine, separation of lame sheep, culling breeding sheep that become lame twice or more in a year, breeding for resistance and vaccination (6, 7).

In 2011 the Farm Animal Welfare Council, an advisory body to GB governments, set a target to reduce the average national lameness prevalence from 10% in 2011 to 5% by 2016 and to <2% by 2021 using existing evidence (8). By 2013, approximately 40% of farmers had stopped routine foot trimming, 51% of farmers were using prompt treatment (6), and the average national lameness prevalence had reduced to 5%. However, many farmers still considered prompt treatment difficult to implement and preferred whole flock management practises such as routine foot trimming and footbathing, despite also reporting that these practises were ineffective in control of footrot (2). Farmers report that catching sheep is more difficult at certain periods of the production cycle, e.g., mating and early lactation and not possible in some pasture situations, e.g., when electric fences are used to contain the flock (2, 7). There is also evidence that lack of knowledge of the most effective managements is associated with not using prompt treatment (9). By 2015 the percentage of farmers using prompt treatment had fallen to 29%, and there was an increase in the prevalence of lameness (7). Consequently, farmer behaviour still needs to change so more farmers use effective managements to treat and prevent lameness in sheep.

One route to influence change is to educate sheep farmers directly. This is currently done through farm talks, events at shows and markets, written materials, and websites as well as flock health clubs (10–12). However, many of the ~70,000 sheep farmers in GB do not engage with any of the above activities (2). Many young farmers attend agricultural colleges, which might be a route to educate farmers and change practice over time, however, most agricultural students are convinced that traditional managements, such as foot trimming and footbathing, are effective to control lameness despite evidence to the contrary (e.g., 6) and they do not change their belief at college (13).

Veterinarians could also influence sheep farmers. Prompt treatment includes use of parenteral antibiotics which must be prescribed by the veterinarian caring for the sheep. This should result in the veterinarian inspecting the flock regularly and an opportunity to discuss approaches to manage lameness in sheep. However, general practitioners typically consider themselves insufficiently knowledgeable to work confidently with sheep farmers (14) and sheep farmers do not consider veterinarians sufficiently knowledgeable to advise on flock health (10). Post graduate training of veterinarians on flock health issues including lameness (e.g., (15)) has led to more competent sheep veterinarians and ‘flock health clubs’ where sheep farmers come together for regular veterinary advice from a knowledgeable practitioner (11, 12). However, veterinary expertise in lameness in sheep is still not consistent across GB.

Lame sheep are present in every flock (16) with prevalence from <1% to >15% (6, 17) and given impact on production, health, and welfare of sheep it would be ideal for all veterinarians to be competent to advise farmers on management of lameness. As with farmers and agriculture students, if all new graduate veterinarians were competent to advise clients on management of lameness, the management of lameness would improve over time as veterinarians entered the industry. Information on management of lameness has been widely disseminated in peer review and professional literature (18–20) so lecturers at veterinary schools might be well informed and consequently students educated appropriately.

In 2018 we carried out research to explore the potential usefulness of new digital learning resources on lameness in sheep for GB veterinary students. As part of this project, we conducted interviews and focus groups with lecturers and students at GB veterinary schools to understand more about current teaching and learning practices. We believe these data provide useful insights to inform future education about lameness in sheep and therefore in this paper we present our findings in relation to: (i) the current beliefs of lecturers and students on treatment and prevention of lameness in sheep, (ii) how students are taught about lameness in sheep and the challenges lecturers and students encounter, and (iii) whether students are competent to advise sheep farmers on management of lameness on graduation.

## 2. Methods

### 2.1. Identification of and interviews with lecturers

Lecturers were selected by purposive and snowball sampling (21). Inclusion criteria were staff at GB veterinary schools (*VS*) who were teaching or had taught sheep medicine to veterinary students. We aimed to interview at least one lecturer from each of the eight *VS* present in GB in 2018. In total 20 lecturers were contacted and 10 participated including one from VS7 who had retired the previous year (Table 1).

**Table 1:** Lecturer and student participants by GB veterinary school and students’ year of study, sex, and interest in working in farm practice.

Veterinary school code	No. of lecturer participants	No. of student participants	Students’ year of study (no.)*	No. of students interested in farm practice	No. male/female students
VS1	1	7	4th	1	1/6
VS2	1	7	4th (5) and 5th (2)	6	3/4
VS3	1	0	–	–	-
VS4	1	9	5th	8	2/7
VS5	3	8	4th	5	3/5
VS6	1	0	–	–	–
VS7	1	0	–	–	–
VS8	1	0	–	–	–

No, number; VS, veterinary school, *4th year students were just beginning their final year clinical rotations. 5^th^ year students had completed their final year rotations and were about to graduate. - = no focus group run at that veterinary school.

Lecturers were informed that participation in the study was voluntary. They were asked to complete a consent form and email it to RC before the interview. The interviews were conducted by RC between February and July 2018 and lasted 20–40 min. They were conducted by telephone, except for one conducted in person when RC was at the *VS* conducting a student focus group. The interviews were semi-structured with a question guide which included prompts (Supplementary material). Topics of discussion were: (i) lecturers’ beliefs about treatment and prevention of lameness, (ii) factors influencing their students’ beliefs, (iii) the methods used to teach students about lameness, and (iv) the challenges around teaching students about lameness in sheep.

### 2.2. Recruitment of students and process for student focus groups

Lecturers at four *VS* organised a student focus group of 7–9 students at their school (Table 1). At VS1, VS4, and VS5 the focus groups were run during clinical teaching time and students who were on farm animal clinical rotation were invited to participate. At VS2 the focus group was an evening event for the ‘student farm animal society’ and all members of the society were invited to participate. Students were provided with the project information letter and attended their focus group voluntarily; they signed a consent form before the focus group began. To encourage participation students were offered refreshments and a short seminar on lameness in sheep after the focus group.

Student focus groups were conducted by RC with a facilitator (KB on three occasions and HN on one) who greeted late arrivals and handed out materials used during the discussion. Topics in the discussion guide (Supplementary material) were: (i) students’ beliefs about treating and preventing lameness in sheep, (ii) approaches to managing lameness in sheep that students had seen on farms and in veterinary practice, (iii) the role of veterinarians on sheep farms, and (iv) students’ experience of learning about lameness in sheep at VS. Focus groups lasted between 50 and 90 min.

### 2.3. Analysis of data from interviews and focus groups

RC and KB performed qualitative content analysis using the Framework Method as described by Gale, Heath (22). Audio recordings of interviews and focus groups were transcribed by an external company (Penguin Office Services, UK). Initially transcripts were checked for accuracy, and to enable researchers to become familiar with the content. A broadly deductive approach using directed qualitative content analysis (23) was used because there were specific, predefined areas of interest. Predefined codes were identified in order to answer specific research questions, for example ‘Are veterinary students aware of recommended practice for treating footrot in sheep?’. Open coding was also done on all transcripts (22) to ensure that important aspects of the data were not missed. Codes were then reviewed and organised into broad categories to form an analytical framework. RC coded all transcripts in NVivo v12.6 (QSR International) and then summarised data by charting into a matrix in Excel (Microsoft). Interpretation of the data and ongoing reviewing and refining of codes were conducted by RC and LG.

## 3. Results

### 3.1. Lecturers’ and students’ expertise and experience with sheep

We interviewed one lecturer at VS1-4 and 6–8, and three lecturers (5a, 5b, and 5c) at VS5, a total of 10 lecturers (Table 1).

Seven lecturers from five *VS* had done research on lameness in sheep. Four of these seven had specialist qualifications (European Diploma of Small Ruminant Health Management or Royal College of Veterinary Surgeons Certificate of Advanced Veterinary Practice). Seven lecturers from seven *VS* had a doctorate degree. Nine lecturers from seven *VS* had worked in first opinion practice (mixed or farm animal) earlier in their careers.

Students were either at the end of their 4th (*n* = 20), or 5th and final (*n* = 11), year of study (Table 1). There were more female than male student participants (Table 1) which is representative of the wider student population at GB *VS* (24). Four students had grown up on farms where sheep were the primary enterprise and three students where sheep were an additional enterprise, e.g., over-wintering lambs. A further 20 students had worked on sheep farms whilst they were at school, only four students had no experience of sheep farming before they started their veterinary degree. The number of students interested in farm animal practice varied by group (Table 1); overall 20/31 (60%) students were interested in farm animal practice; again this aligns with the wider student population (24).

### 3.2. Current beliefs of lecturers and students on treatment and prevention of lameness in sheep

#### 3.2.1. Lecturers’ beliefs and practices for management of lameness in sheep

All the lecturers stated that research evidence influenced the way they would advise farmers to manage lameness.

*Lecturer VS5c: I would say peer review papers, that would be one of the main [sources of information]. I do then look in websites, so for example, it could be the Sheep [Veterinary] Society, again if we are talking specifically for sheep, AHDB [Agriculture and Horticulture Development Board, GB sheep industry levy body] for example, the Moredun [Foundation].*

##### 3.2.1.1. Treatment of footrot

All lecturers stated that sheep with footrot should be treated with systemic antibiotics and that foot trimming should be avoided, this is in agreement with research evidence (5).

##### 3.2.1.2. Control of lameness

Footrot is the dominant cause of lameness in GB and lecturers generally focused on footrot when discussing control of lameness. Most lecturers, but not all, focused on reducing spread of footrot through prompt treatment of diseased sheep (3), combined with quarantine of incoming sheep, separation of diseased sheep, and culling of repeatedly lame sheep. Lecturers proposed vaccination against footrot in some situations, primarily where prevalence of lameness was particularly high as a route to gain control of lameness. These are the key flock managements with evidence of efficacy: quarantine and separation of lame sheep are strongly associated with reduced prevalence of lameness, culling persistently, or repeatedly, lame ewes reduces prevalence of footrot, and vaccination for >5 years reduces prevalence of lameness (6, 7, 17, 25).

*Lecturer VS5c: I think the quarantine would be one of my main [points], just not to introduce any disease in the flock. I would advise on early diagnosis, I think that would be the other main point, so try and find the lame sheep as soon as possible and then if they are lame and, you know if it looks like there’s an infectious cause then do the protocol that I’ve said before, so the single treatment, isolation of the animal, that would be another big point. I would then I think discuss methods that [the farmer is already using] to deal with lameness or if he is doing footbathing and have a look at the facilities, find out if he’s trimming* [the tone indicated Lecturer VS5c thought that this was a negative management practice] *and then potentially talk about vaccination.*

However, many of the lecturers also recommended footbathing. Footbathing is a traditional practice that many farmers still use, despite evidence that flocks that are footbathed have higher prevalence of lameness than those that are never footbathed (6, 25, 26). Lecturer VS8 (below) recommended footbathing to prevent lameness, despite commenting that it is unlikely to be effective, an example of cognitive dissonance. Farmers also demonstrate cognitive dissonance around footbathing, probably because it is considered easier to implement than catching individual lame sheep for treatment (2).

*Interviewer: can you describe how you would recommend preventing lameness in sheep?*

*Lecturer VS8: so depending on the flock circumstances either vaccination, or, well, footbathing but I know a lot of shepherds aren’t particularly great at footbathing their sheep, but trying to get a footbathing regime, trying to dissuade them from any routine foot trimming that they do, just go to foot checks rather than trims. And then potentially field management, field rotation depending on what it is they are dealing with.*

Lecturers frequently stated that there was no ‘one solution fits all’ to manage lameness on sheep farms and they would advise a farmer based on the problem, the production system, current management practices, and what they considered the farmer was willing or able to do. Four lecturers referred to the Five Point Plan (27), an approach to managing lameness that has been widely recommended by GB industry bodies (e.g., 28, 29). These lecturers described how they would use the framework of the Five Point Plan as a guide, and then focus on key areas for implementation, depending on the particular characteristics of the farm in question.

*Lecturer VS3: I base my preventative teaching round relevant aspects of the Five Point Plan, so I would go through all of those aspects, but then tailor it to individual farms within that framework.*

One of the four lecturers commented that the Five Point Plan overcomplicates the explanation of management practices that are effective to control lameness.

*Lecturer VS5b: as with a lot of topics the complexity makes it difficult, so the Five Point Plan is useful because it has five main points, but actually those points are quite complex. Some of them are quite complex in themselves, and there’s some that I can’t even remember what they are called because I can remember what you are meant to do but not how it’s categorised.*

Sheep farmers believe that all sheep flocks are ‘different’ (10) which creates a perception that sheep flocks are complex and that whilst farmers are highly knowledgeable of their flock, veterinarians ‘must’ have expertise of their flock to advise them. Many veterinarians also believe that sheep flocks are complex (14, 30) which could contribute to their lack of confidence when advising sheep farmers. Interestingly, an elicitation study with expert sheep advisors indicated that they had standard recommendations when advising sheep farmers on management of lameness and did not view sheep flocks as fundamentally different from each other (31); they had self-confidence as experts, and they had the confidence of their clients.

#### 3.2.2. Students’ beliefs and practices for management of lameness in sheep

##### 3.2.2.1. Treatment of footrot

The majority of students stated that they would treat individual cases of footrot using systemic and topical oxytetracycline (antibiotic) and would avoid foot trimming.

##### 3.2.2.2. Control of lameness

Students did not refer to prompt treatment as the best management practice to control spread of footrot, the dominant cause of lameness in GB.

Foot trimming was discussed in all the student focus groups. Students had variable knowledge of the evidence on foot trimming. Most students knew that foot trimming sheep with footrot delays recovery (5), but some were unaware that routine foot trimming damages feet (6, 32) and increases prevalence of lameness (e.g., 6, 7). This is exemplified in group VS2 below who were unaware that routine foot trimming is harmful and believed that the main risk from foot trimming was poor hygiene.

*Group VS2*

*Student A: for some of these commercial flocks, with a couple of thousand ewes, the logistics of doing a routine trim and trimming everything and leaving the lame ones to get back [at a later date], when they are in amongst a couple of thousand ewes, you’re never gonna find ‘em, it’s never going to happen.*

*Student H: I agree with [Student A]. We’ve got lots of hill flocks in Wales and you might just have a flock in once or twice a year [for routine foot trimming] so they’re not gonna leave some and then get them back in to just do the odd one or two.*

*…*

*Student K: I always thought that the reason they say not to trim is because people don’t have a disinfectant, they put the trimmers between [sheep] so they spread it throughout the flock. Whereas if you did do something like that and properly disinfect it then probably trimming isn’t… too bad.*

Group VS1 had the best understanding of the evidence around routine foot trimming, stating that “*there’s no real evidence that routine foot trimming has any major effect really … but badly done it causes problems*.”

Students also suggested a range of measures to control lameness in sheep that have evidence of efficacy including culling, quarantine, vaccination, and separation of lame sheep. However, all student groups proposed footbathing to control lameness. Some were taught about footbathing in detail in lectures (as shown in the quote below), others from farming backgrounds had used footbaths on their own farms.

*Group VS4*

*Interviewer: what would you consider in terms of preventing lameness if you were advising the farmer?*

*Student L: [We] talked about footbathing a [little] bit, so we discussed in lectures whether that was 10% zinc sulphate or 3% formalin, and yeah, getting them out onto a dry standing, trying to have some clean grazing areas.*

*Group VS5*

*Interviewer: What would you consider advising a farmer if [they were] interested in preventing footrot?*

*Student J: Closing the flock and if you were buying in, isolate [quarantine].*

*Student W: Culling.*

*Student A: If [the sheep] have more than one [case of lameness], mark the ones that [are affected] and then if they’re chronic cases cull them out.*

In summary, whilst lecturers stated that they followed evidence-based practice, some of their teaching did not have a strong evidence base, e.g., footbathing, and they believed that prevention of lameness required a flock specific approach. The majority of students were confident in how to treat cases of footrot but most had incomplete understanding of the evidence regarding control of lameness.

### 3.3. Teaching and learning about lameness in sheep at GB veterinary schools and the challenges lecturers and students encounter

There was significant variability between the *VS* in both the quantity and type of teaching on lameness in sheep. All students received lectures on lameness in sheep. At VS1 and VS3 there were 3 h of lectures whilst at other *VS* there was a maximum 1 h of lectures, with one *VS* offering 15 min of lecture time. At five of eight *VS* students had practical classes (clinical examination of live sheep or with feet from cadavers) and at four of eight *VS* students had self-directed or problem-based learning exercises on lameness in sheep.

#### 3.3.1. Limited curriculum time on diseases of sheep

Four lecturers considered that the time allocated within the curriculum was not sufficient to teach students about lameness in sheep. Lecturers attempted to mitigate for this through self-directed learning and small group tutorials, or by prioritising teaching on common conditions of sheep including lameness. The amount of time on lameness was often still limited, for example Lecturer VS4 described increasing the amount of time teaching on lameness from half a lecture (25 min) to one lecture (50 min). Several lecturers reported that they targeted detailed teaching on lameness in sheep to elective students, who chose to take additional training in farm animal medicine.

*Lecturer VS2: The problem is for our undergraduates a third of a lecture [20 min] is stretching it when you have everything else to cover. So it’s just really picking out if there are any real big changes that are gonna affect them and thinking from a perspective of day one skills. You know it really is that basic.*

Two of the lecturers also mentioned that sheep receive low priority within the veterinary curriculum, and this was a view shared by many of the students who reported that sheep were often ‘skipped over’ [Group VS1] on the ruminant course, with less lecture time on sheep lameness than cattle lameness, and practical teaching on sheep lameness included within a cattle practical, or not occurring at all.

*Group VS2*

*Student K: I think we had one lecture on [lameness in sheep].*

*Student O: That’s the thing, had one lecture, on all of the lesions, isn’t it?*

*Student K: We barely touched on it, we had like 5 lectures for cattle.*

Whilst Lecturer VS8 highlighted that the low priority given to sheep teaching was something the *VS* was trying to address, Lecturer VS5a argued that a relatively small number of students would enter sheep practice and so it was not justifiable to increase the time for teaching about lameness in sheep.

*Lecturer VS8: It has been raised at work that sheep are often shoe-horned in at the end of ruminant lectures, which I don’t think’s uncommon between the vet schools really. But I know X [colleague] is trying to redress the balance a little bit.*

*Lecturer VS5a: If one wanted to up the quality of the teaching one would probably make the problem-based learning [about lameness in sheep] across the whole year and also discuss it again in tutorials. But again, I struggle to justify that when probably no more than 20 of the students are actually going to see a reasonable number of sheep in their careers.*

#### 3.3.2. Limited clinical experience on sheep

A challenge frequently reported by students was the limited opportunity to gain clinical experience of lameness in sheep. They reported that they were unlikely to see sheep during clinical extra-mural study (EMS) placements apart from emergency procedures around lambing, and highlighted that veterinarians are unlikely to attend a flock because of a lameness issue.

*Student S (VS5): [I have] done quite a lot of farm EMS but thinking about it in terms of lameness, the only time we have done sheep has been caesareans and that’ll be it.*

For many students there were also limited opportunities to see cases of lameness whilst on clinical rotations at their *VS* because of the *ad hoc* nature of clinical teaching. The students who had received more proactive clinical teaching on lameness were positive about the benefit of this experience.

*Student G (VS2): If you have a case on one of your clinic days you might go, you’d obviously go with them and do things like that, but we never really have any specific practical teaching on identification of lesions or things like that.*

*Student J (VS1): I really enjoyed the day that we had … actually touching the sheep! < Laughter > Seeing real live cases. ‘cause there was one of them which was lame and we had to catch it and then he [the lecturer] left it to us, [he said], “What would you do?” And we had to say what we thought were the best treatment options, and even stuff like, so they have the little sebaceous gland, and we got confused, we thought that was a lesion, but –<Laughter > But it wasn’t.*

The benefits of seeing clinical cases were recognised by both lecturers and students; benefits included developing practical skills, putting theoretical information into context and improving their ability to remember information. A common topic across conversations with students and lecturers was the difficulty in learning to diagnose common causes of lameness from one photograph of a foot lesion with ‘classic’ pathology. They said that in reality there are stages of disease progression, often more than one type of lesion on a foot, and that contamination of feet with, e.g., mud made diagnosis difficult.

*Student A (VS5): The way we were taught it was like … I think in the lectures basically just like different pictures, this is this, this is this, this is this and it was the same with the practical. I think you had to link the picture to the [disease name], but obviously from a picture, sometimes you cannot orientate yourself which way up is a foot or whatever, and then also they’re not all gonna look like that.*

*Student M (VS2): I don’t remember anything I’ve been taught in a lecture theatre really, but it’s seeing the cases and actually doing something, you get way more involved with it I think.*

#### 3.3.3. Students’ previous experiences of foot trimming in sheep

Five of the ten lecturers expressed concern that prior beliefs that students had from working on sheep farms or in their early years at *VS* made them resistant to believing the current evidence on management of lameness in sheep. In particular, the practical and immersive nature of lambing placements (typically done in year 1) resulted in students remembering practices they had seen on farm better than those taught at the VS.

Students and lecturers reported that foot trimming was taught in VS4 and VS5, although students at VS5 were taught situations when foot trimming was not appropriate. Lecturer VS1 also highlighted that some non-veterinary staff in VS1 were still teaching students to trim sheep feet despite research evidence.

*Lecturer VS5b: [one] problem we have with students is that what we teach them is very different to what they see when they go on farm or into practice. So when I’m trying to get them to talk about how you treat lameness, the answers I get back are what farmers are doing on farm, not what they should be doing. Because it’s practical they remember it so much better than what they were taught in a lecture, so I think one of the real difficulties is getting them to learn the difference between what’s done and what should be done.*

Many of the students also commented that they had either observed or carried out foot trimming during lambing placements on farm. However, despite their lecturers’ concerns, most said they later became aware that foot trimming is detrimental (3, 5) and changed their beliefs and would not recommend or use foot trimming. A small number of students from sheep farming backgrounds believed foot trimming was appropriate despite research evidence that it is detrimental; this was also observed in agricultural students from farming backgrounds (13).

*Student F (VS4): So I think I just learnt [foot trimming] off the farmer, ‘We make it bleed, we make it bleed and it’ll run away sound the next day’ and then subsequently have had clinical teaching and have been told that there’s an evidence base behind to say that that’s actually harmful, so happy to, to not do that anymore.*

*Student A (VS2): Similar to [Student R], at the moment we would correctively trim. I know now from coming to university it’s not best practice but at home at the moment we would correctively trim it, we would yeah, your blue oxytetracycline spray, formalin bath to correct.*

*Group VS4*

*Student F: We trimmed feet on that practical I think.*

*Student L: I literally think [name] just handed us a trimmer [and said] “whilst you tip the sheep, why don’t you trim its feet?”*

In summary, teaching time and opportunities for students to gain clinical experience of lameness were very limited therefore many students graduate with little clinical experience of lameness in sheep, despite it being one of the most common (6) and economically important (4) diseases on GB sheep farms. Students are still exposed to foot trimming but despite their lecturers’ concerns, most students believed their lecturers’ teaching over their experiences elsewhere. However, students who were from farming backgrounds were more recalcitrant to changing their beliefs.

## 4. Discussion

The aim of our research was to investigate whether new graduate veterinarians are competent to advise sheep farmers on control of lameness and so contribute to reducing the prevalence of lameness in sheep to <2%, the figure proposed by FAWC (8). There were two key findings from our study: first, students did not have a good understanding of the evidence behind the various management practices used for lameness and therefore could not prioritise the most effective managements. Second, most students had not gained clinical experience of lameness in sheep prior to graduation.

Lecturers and veterinary students proposed a range of practices to control lameness, and whilst most lecturers were aware of the evidence behind these practices, students were not. Overall, students could not prioritise the management practices they had been taught, often focusing on footbathing for which there is no evidence of efficacy, rather than prompt treatment, which alone can reduce lameness prevalence to <2% (3).

Some lecturers used the ‘Five Point Plan’ as a guide, although as highlighted it includes about 20 management practices within the ‘five points’ without prioritisation which adds to the complexity of managing lameness. Lecturers said they would prioritise management practices based on the specific flock and the farmer, rather than the strength of evidence for their efficacy.

The belief that management of lameness requires flock specific approaches and the failure to prioritise the most effective practices contribute to making advice on management of lameness in sheep unnecessarily complicated. A consistent message to stop footbathing, stop foot trimming, use quarantine, use prompt treatment, and consider vaccination against footrot (‘vaccination can be effective over time’), would enable lecturers and students to prioritise a few effective management practices and to advise farmers confidently. It would also prevent veterinarians and farmers selecting management practices that they prefer because they are convenient even if they are not effective (2, 30).

Lecturers and students considered that there was insufficient time to teach sheep health. One option employed by some lecturers was to prioritise teaching what they considered the common health issues, including lameness, rather than giving equal teaching time to all areas of sheep health. This was particularly evident at VS1. Rationalising teaching of sheep health to those conditions that all veterinary graduates are likely to see would be a useful way to make most effective use of the time available for sheep teaching and would ensure omnicompetence across species as required currently. Small animal practitioners have been consulted on core competencies for orthopaedic diseases (33) and a similar consultation with sheep practitioners to identify core competencies in sheep health is one option to improve competency in new graduates to manage lameness in sheep.

Lecturers and students reported that there was insufficient opportunity for students to gain clinical experience of lameness in sheep. Observing and engaging in clinical situations enables students to develop practical skills and increases their confidence and motivation, strengthens and deepens theoretical knowledge, and increases retention of information (34, 35). EMS placements are a requirement for students and important for developing communication, interpersonal, and technical skills (36). EMS placements could be better utilised to provide students with clinical experience in sheep lameness. There are over 70,000 sheep flocks in GB and most have lame sheep (7, 16) and, indeed, many *VS* have sheep flocks with lame sheep. One option would be for students to spend some of their allocated clinical EMS placements on a sheep farm, supervised by a veterinarian. They would develop practical skills in diagnosis, treatment and control of lameness and other flock health issues such parasitic disease and flock improvement through better nutrition and breeding. Students could discuss and reflect on their experiences with their supervisor during their placement. This experience could help students to develop communication and interpersonal skills with sheep farmers and address the current initiatives to increase interaction between sheep farmers and veterinarians (11, 12). Whilst it might not be possible for all students to undertake farm experience, it could be offered to those with a particular interest in farm animal practice. Alternatively, one *VS* could offer a specialisation in sheep health for students from all GB VS. This is done for dairy cattle in the United States of America where the National Center of Excellence in Dairy Production Medicine Education for Veterinarians (37) offers an eight-week intensive rotation to veterinary students from around the world, to provide students with knowledge and skills relating to dairy herd health planning. A similar approach could facilitate expertise in sheep flock health planning and management in GB.

Another approach to improve students’ knowledge of lameness in sheep would be online clinical learning environments and simulated clients (e.g., 38, 39–41) during clinical rotations. This might be a more practical approach for larger numbers of students to engage with sheep health in a clinical context, however, no students mentioned these novel learning technologies and their benefit to increase confidence in managing lameness in a real clinical setting is not known.

These ideas are not a definitive list of recommendations, rather a starting point to exploring ways to increase new graduates’ knowledge of and confidence in management of lameness. Whilst none of the above might be considered acceptable to a *VS*, there are lame sheep in most flocks most of the time and the tools to control lameness are known, so ensuring new graduates are competent to advise farmers should be possible.

## 5. Conclusion

Lameness is a common and economically important disease of sheep in GB with lame sheep on almost every sheep farm. GB veterinary students, even those who wish to specialise in farm animal practice, are graduating without confidence or competence to advise farmers on management of lameness in sheep. Key challenges to learning about lameness in sheep were insufficient teaching time available for sheep health within the veterinary curriculum, and the limited opportunities for students to gain clinical experience with sheep. Finding ways that students can gain in-person clinical experience with sheep through EMS placements or alternative teaching approaches would address this challenge and ensure veterinary graduates are competent to manage lameness in sheep.

## Data availability statement

The datasets presented in this article are not readily available because they contain information that may facilitate identification of participants. Requests to access the datasets should be directed to RC, rachel.clifton@nottingham.ac.uk.

## Ethics statement

The studies involving human participants were reviewed and approved by Biomedical and Scientific Research Ethics Sub-Committee, University of Warwick. The patients/participants provided their written informed consent to participate in this study.

## Author contributions

RC and LG conceived and designed the study. RC and KB collected and analysed the data. RC and LG wrote the manuscript which was approved by all authors. All authors contributed to the article and approved the submitted version.

## Funding

University of Warwick Higher Education Innovation Fund.

## Conflict of interest

The authors declare that the research was conducted in the absence of any commercial or financial relationships that could be construed as a potential conflict of interest.

## Publisher’s note

All claims expressed in this article are solely those of the authors and do not necessarily represent those of their affiliated organizations, or those of the publisher, the editors and the reviewers. Any product that may be evaluated in this article, or claim that may be made by its manufacturer, is not guaranteed or endorsed by the publisher.
