# Supplementary material for: “I didn’t see a sheep”: perspectives of lecturers and students at veterinary schools in Great Britain on learning about lameness in sheep
Source: Front Vet Sci. 2023 Jun 9;10:1171853. doi: 10.3389/fvets.2023.1171853 (PMC10288135; doi:10.3389/fvets.2023.1171853)
Supplement: Supplementary file 1 [file Data_Sheet_1.PDF]

"I didn't see a sheep": perspectives of lecturers and students at veterinary schools in Great Britain on learning about lameness in sheep – Supplementary information

Question guide for interviews with veterinary lecturers

Introduction

Thank you for agreeing to participate in an interview. The aim of this project is to understand current approaches to managing lameness in sheep and teaching in this area, and to develop a new online resource for veterinary students, lecturers and practitioners. The project is funded by the [organisation]. This interview should take no more than half an hour.

Opening questions

Background and course structure

1. Can you tell me a bit about your own background?
   - *Experience of working in practice/in research/in vet schools*
2. Can you tell me about the courses you teach?
   - *How is the teaching structured at the vet school? Preclinical and clinical or more integrated from the start?*
   - *Where does lameness get covered? How much teaching time is dedicated to lameness?*
   - *How many colleagues are involved in teaching?*

Core part

Managing lameness and foot health

1. Can you describe to me how you would treat an individual sheep with footrot?
2. Can you describe how you would recommend preventing lameness in sheep?

For both questions:

   - *Tell me about your reasons for that*
   - *Can you tell me about an occasion when you changed your beliefs about treatment/prevention of footrot?*

3. How do you think what you have outlined relates to what is done on sheep farms?
   - *If there are differences – what are the reasons for the differences? How could this be improved? Can you describe examples of change?*
   - *If no differences – can you give me some examples? Can you describe how this has changed?*

Teaching

1. Can you tell me about how you see your role as a lecturer?
   - *What do you aim to achieve? What are your responsibilities?*
   - *What is their perspective on their role as a teacher? Are they focused on students passing assessments, benefit for the individual students, benefit for profession? Do they see their role in a wider context?*
2. Can you tell me about how you teach students about lameness in sheep?
   - *Could you give me examples about what different methods help you achieve when you are teaching?*
   - *Can you describe any ways in which you have changed your teaching?*

- *What influences your choices of which teaching methods to use? Is this purely practical, do they read literature/guidance on learning styles etc?*
- *What are the challenges for teaching this subject?*

3. Could you tell me about where you find evidence/information for your teaching?
   - Can you describe how you prepare for a lecture?
   - Is material updated regularly?
   - Do they use their own knowledge or literature search etc?
4. Where else do you think students pick up messages about managing lameness in sheep?
   - *Sheep farms/seeing practice/reading literature*
   - *What do you think influences their beliefs? Which sources do you think they place most value on?*
   - *What role do lecturers play in this? Do they feel they have any influence in how students view information?*

Provision of new resources

1. Can you describe anything you would change about how vet students are taught about lameness in sheep?
   - *Change in approach to teaching – skills vs clinical knowledge? Change in content? Change in teaching method – theory/case studies/practical?*
2. With regards to new resources about lameness, what could be provided that would help you or your students?
   - *images*
   - *interactive sessions*
   - *video clips*
   - *materials for self study*
   - *case studies/examples*
   - *evidence based/research data – regular updates*
   - *Can you give examples of how these resources would be useful?*
3. Can you give examples of resources that would be useful for vets working in first opinion practice?
   - *CPD*
   - *images*
   - *interactive sessions*
   - *video clips*
   - *materials for self study*
   - *case studies/examples*
   - *evidence based/research data – regular updates*
   - *Can you give examples of how these resources would be useful?*

Winding down

1. What do you think are the future challenges for managing lameness in sheep?
2. Is there anything else that you feel is important that we haven't already covered?

Thank you for your participation and your time. Would you like to be kept informed about the development of the online resource?

Question guide for focus groups with veterinary students

Introduction

Hi everyone, I'm [name] and this is my colleague [name] who is helping me. We are both from [organisation]. We are working on a project to design an online resource for vet students and lecturers. We would like to find out about your opinions about lameness in sheep, and what would be useful to you as part of an online resource.

You should have received an information letter, which explained that we would be recording the discussion. This just makes it easier for us to look back at what has been said, and everything will be anonymized. If you are happy with this, please could you sign the consent form in front of you and pass it back to us.

Ok great so we can get started with the discussion. It is important to remember that it doesn't matter how much you know about this topic or how much experience you have, we are interested in your opinions. There are no right or wrong answers to our questions, and if you have different opinions from each other that's fine. Feel free to volunteer answers at any point during the conversation and to discuss things as a group as this will really help us get a better understanding.

It is however important that everyone has a chance to share their opinions, and for the recording it is easier if you don't talk over each other, so I will make sure everyone has time to contribute before we move onto the next question.

Introductions
Ok, so for the recording we need everyone to introduce themselves and say a couple of sentences. This just makes it easier for us to follow when we listen back to the recording.

So please could you introduce yourself using the guide we have handed out.

I will go first as an example…

Main questions

1. Can you tell me a bit about your areas of interest?
   - What type of practice do you think you want to go into when you qualify?
   - Tell me about the courses that have interested you the most?
2. Can you tell me about how much you have covered lameness in sheep in your course so far?

3. Can you describe to me how you would treat a sheep with footrot?
   - Tell me about your reasons for that
4. Can you describe how you would recommend preventing lameness in sheep?
   - Tell me about your reasons for that
5. Can you tell me about the approaches to lameness management you have seen used by vets in practice?

- Is this different to what you have been taught?
- Can you give me some examples of the differences?
- If so, why do you think there are differences?

6. Can you tell me about the approaches to lameness management you have seen used on sheep farms?
   - Is this different to what you have been taught?
   - Can you give me some examples of the differences?
   - If so, why do you think there are differences?
7. Can you describe how you see the role of vets on sheep farms?
   - Preventative, flock health, emergencies
   - Prompts: what do you think sheep farmers use vets for? What do you think vets can contribute on sheep farms?
   - What do vets need to consider when working with farmers to manage lameness?

8. Can you tell me about the teaching you have had about lameness in sheep during your course?
   - Teaching method e.g. practical, lecture, interactive, assignments, trips
   - What did you enjoy most about learning this subject?
   - Can you give any examples of how it could be improved?
   - Can you give some examples of teaching you had during another subject that you found particularly helpful?
   - What do you find difficult to learn about this subject?
9. If you had a project to do on managing lameness in sheep, where would you look for information?
   - Internet, library, journal articles
   - If different resources gave you different information, how would you approach this?
   - Can you give me an example of where you have had to research information like this? How did you find the process?
10. If we were to provide an online resource about lameness for you to use, what do you think would be the best format for this?

Ok so we have some example materials to look at on the iPads. There are 6 examples and they are all shown on the web page that is open, they are labeled A-F. E and F are both pdfs. There are two videos, a quiz and a case study. You don't need to do all of each one but just get an idea of what it's like.

Take a few minutes to have a look at these, and you can try answering the questions and watching the video. Please think about what you like and don't like about them, then write your two favourites on separate pieces of paper. It doesn't matter if you think differently to other people, we will mix all the answers up in the middle of the table.

Give out iPads

So [resource] seems to be popular – can you tell me what you liked about this one? Was there anything that you didn't like or could be improved?

What was it about [resource] and [resource] that you didn't like? Was there anything that was good about these?

Alternatively:
We have quite a mix of opinions, so lets start with [resource]: what did you like about this one, and what wasn't good? etc

- When would you prefer something that you could do in short sections, or something more like a full lesson?
- What kind of device would you like to be able to access information on?
- So at the start of the video it says 'My name, vet'. Does this make a difference to you? What type of person do you think should present the information?
- When do you prefer cartoons, tests, real life etc?

Prompt: has everyone contributed to answering the question?

Summary
So that brings us to the end of the questions, but before we end I would just like to summarise what we have talked about and check there isn't anything else you would like to add.

- In terms of treating lameness…
- In terms of what you enjoy about your current course…
- In terms of what you would like from a new learning resource…
